# Evaluating the synergy: anxiety prevalence and alcohol consumption patterns in high-income countries using Granger causality analysis

**DOI:** 10.1186/s12889-025-21402-6

**Published:** 2025-01-20

**Authors:** Binguni Senarathne, Dinithi Palliyaguru, Anne Oshini, Janudi Gamage, Ruwan Jayathilaka, Lochana Rajamanthri, Colinie Wickramarachchi

**Affiliations:** Sri Lanka Institute of Information Technology, SLIIT Business School, New Kandy Road, Malabe, Sri Lanka

**Keywords:** Anxiety prevalence, Alcohol consumption, Anxiety disorder, Granger causality, High-income countries

## Abstract

**Background:**

Alcohol consumption frequently experiences episodes of severe anxiety. This study set out to explore the long-term effects of alcohol consumption on anxiety, revealing insights into how alcohol consumption uniquely impact anxiety, aiming to inform mental health and public health approaches. This research paper explores the complex relationship between the prevalence of anxiety and the consumption patterns of wine, beer, and spirits across fifty-two high-income countries with a continental analysis.

**Method:**

By employing significant secondary data taken from the World Health Organization and Our World in Data online databases and covering the period from 1990 to 2019, this study seeks to explore various causality relationships over this period. Its primary objective is to fill the empirical gap in existing research by using Granger causality analysis to reveal the dynamic relationships between the consumption of alcohol and the prevalence of anxiety. This study endeavours to provide a continental analysis of the high-income countries, which allows for including a comprehensive viewpoint in the context of a single investigation.

**Results:**

The findings demonstrate a variety of patterns of causality in alcohol consumption and anxiety prevalence in a one-way causal relationship across specific continents, a bidirectional relationship in others, and no apparent causal relationship in several countries.

**Conclusion:**

The inference made from the study’s results goes beyond scholarly curiosity; it establishes the foundation for further investigations and the development of customised policies aimed at reducing the mutually reinforcing dangers of alcohol consumption and anxiety disorders.

**Supplementary Information:**

The online version contains supplementary material available at 10.1186/s12889-025-21402-6.

## Introduction

Alcohol has been an essential element of human culture and social interaction for centuries, serving different community functions [1, 2]. However, its use has significant health consequences, thus of great interest to researchers, legislators, and public health experts. It considers alcohol consumption under three main categories: wine, beer, and spirits - and its complex effect on anxiety in high-income countries where anxiety prevalence is highest through a continental analysis. Alcohol consumption customs have evolved with significant variations in different geographies and civilisations. Direct alcohol consumption has been associated with several conceivable well-being preferences. In contrast, moderate or excessive alcohol consumption has been repeatedly associated with adverse effects on physical and psychological well-being.

For a considerable time, psychological and medical research has focused on anxiety, a widespread and complex psychological disorder. Worry, uneasiness, and fear are the hallmarks of stress, which profoundly impact social interaction, physical health, and mental health [3, 4]. Its prevalence across demographics and cultures has been extensively studied, making it a worldwide problem. As anxiety and alcohol consumption serve distinct purposes in different groups and have been widely accepted parts of human culture and social interaction for ages [5, 6], it is essential to understand how they interact to create more effective psychological and public health interventions. The three categories of alcohol consumption habits that were selected, wine, beer, and spirits, are noteworthy for several reasons, particularly when considering the manner of their interaction and their effect on anxiety.

Wine is a beverage and a sophisticated emblem of custom, elegance, and social dynamics. It is frequently honoured for its cultural and historical value. Beyond its complex flavour profile, wine’s effects on physical and mental health have been the subject of much discussion and investigation [7, 8]. Drinking wine in moderation has been linked to several advantageous and disadvantageous health effects, including the ability to reduce stress and the risk of addiction [9]. Beer is a prominent beverage in many countries, as it is one of the oldest and most extensively used types of alcohol [8, 9]. While social rituals, leisure, and relaxation are frequently linked to its intake, its effects on physical and mental health are varied and multidimensional [10, 11]. A contradictory association has frequently been found that in several ways beer intake can affect variables, including anxiety, mood, and social behaviour, according to researchers [7, 12]. Spirits are a class of highly alcoholic beverages prized for their strength and variety of cultural meanings. Spirits have a variety of functions in people’s lives, from festive beverages to coping strategies [7, 13]. An important topic of research is the linkage between alcohol use and mental health, especially anxiety. While some people find comfort and short-term respite in these drinks, others could suffer from increased anxiety and long-term detrimental repercussions.

This study holds significance in four ways, including the fact that it provides essential insights into the intricate relationship between alcohol consumption and anxiety prevalence in high-income countries with a continental approach.

First, the complex relationships between wine, beer, and spirit consumption and the prevalence of anxiety have been extensively studied in research studies [14–16]. While many of these studies have concentrated on different variables, much research has yet to be done on all four variables simultaneously under Granger causality analysis. It is this critical issue that this study undertakes. There is a knowledge lacuna that exists for a comprehensive examination of these associations over an extended period, even despite independent journal articles exploring the impact of alcohol consumption on anxiety under wine, beer, and spirit consumption patterns. Furthermore, relatively few such studies have been conducted for the high-income category, even during shorter periods. To the best of the researcher’s knowledge, this research endeavours as a solution to the information gap by conducting extensive investigations that use a continental approach to examine the various impacts of alcohol consumption on the prevalence of anxiety in high-income countries. As a result, the study’s diligence, encompassing a wide range of variables, countries, and years, adopts a sound methodological helpful approach to other researchers interested in this field.

Second, the results of these investigations were carefully combined using Granger causality analysis, and the causal relationships between variables were graphically shown with arrows. It is interesting to note that, in contrast to all previous research employing Granger causality analysis, these arrows in the study assist in indicating both the existence of a causal relationship and its degree of influence. Consequently, by confirming the stationarity and change of the variables generated for each variable in this study, the lengths of the arrows were adjusted to enhance the graphical representation and emphasise the strength of the established causal relationships. The deliberate fluctuation in arrow lengths depicts each variable’s respective influence, on the others.

Third, the investigation allowed the researchers to examine how all four variables function in diverse countries across six continents. Therefore, this study addresses the need for more comprehensive analysis over longer periods and in high-income countries, offering previously inaccessible fresh data and insights. The investigation results also reveal new factors and patterns that could be investigated in more detail in future research.

Fourth, it offers empirical data to support targeted initiatives and policies that attempt to control alcohol use and treat mental health concerns for legislators and healthcare providers. By clarifying the reciprocal effects between psychological well-being and alcohol consumption, the study contributes to the knowledge of behavioural health economics among scholars and researchers. In addition, this study provides a comprehensive understanding of the variables influencing anxiety and alcohol consumption for the public and individuals who are directly impacted by these problems, which may assist them in making better decisions at the individual, group, and community levels. In conclusion, the study adds to the body of academic knowledge and to enhances societal well-being and public health outcomes by examining the relationships via the Granger causality perspective.

 The remainder of the paper is organised as follows: A review of previous research on the relationship between alcohol consumption and anxiety prevalence is included in Sect. 2. The specifics of the data and methodology are contained in Sect. 3. The empirical results are presented in Sect. 4. The discussion on the topic is carried out in Sect. 5, and the study’s conclusion and policy implications are provided in Sect. 6.

### Literature review

Numerous studies have demonstrated a wealth of evidence showing a bidirectional relationship between anxiety and alcohol consumption [5, 17] and it is estimated that 4.05% of people worldwide suffer from anxiety. Between 1990 and 2019 [18], the number of afflicted people grew significantly, from 194.9 million to 301.4 million, including 58 million children and adolescents worldwide. Twelve thousand five hundred thirty-seven instances of mental health problems per 100,000 people was the global prevalence rate and three thousand eight hundred ninety-five cases of anxiety per 100,000 people are found among them [18]. Excessive concern and fear, as well as associated behavioural abnormalities, are hallmarks of anxiety disorder [4]. The extent to which symptoms are severe depends on how much pain or functional impairment they cause. Anxiety disorders can manifest in many ways. One instance of this is separation anxiety disorder, which is defined by an overwhelming dread or worry over being separated from persons to whom one has strong emotional attachments [19]. The next condition is generalized anxiety disorder, which is typified by excessive concern. Lastly, panic disorder, is characterised, inter alia, by panic attacks. Anxiety is frequently the result of chronically high alcohol consumption [17]. The hallmark of alcoholism is recurrent, high-volume drinking episodes interspersed with intervals of abstinence, which can change brain chemistry and increase anxiety [20]. Anxiety and alcohol consumption are the most prevalent mental health conditions that affect the general population each year, accounting for over 20% of cases [21]. It is theoretically possible for excessive alcohol consumption to trigger the onset of anxiety symptoms via a combination of psychological and biological processes [2, 5, 22]. Therefore, the complex interactions between alcohol consumption and anxiety prevalence emphasise how urgent it is to address these co-occurring conditions because they not only significantly increase the burden of mental health worldwide but also call for all-encompassing strategies that consider biological and psychological aspects to promote well-being and lower the prevalence of anxiety-related diseases.

Alcohol consumption and anxiety prevalence are closely related [6, 22]. For individuals who use alcohol as a kind of self-medication for anxiety [5, 14], it can have positive effects. While alcohol consumption may serve as a communal lubricant that promotes higher-quality social intelligence, alcohol is an anxiolytic that has the short-term benefit of reducing anxiety [20]. Many theoretical models suggest that people drink alcohol to lessen their anxiety symptoms [8, 23, 24]. To the best of our knowledge, people with anxiety may use alcohol as a coping strategy to balance off the adverse effects of their illness, which can eventually result in the development of alcohol use disorders [20]. National household surveys carried out in several African countries have revealed serious concerns about South African women’s drinking habits. The results of these studies show that a significant fraction of South African women who drink alcohol do it at amounts deemed “hazardous.” According to the statistics, 15.6% of South African women who drink belong to the group of heavy drinkers [13, 25], which is defined as those who consume fifteen or more standard drinks in a week. The predominance of dangerous single-occasion drinking, which accounts for 30.5% of female drinkers reporting consuming five or more standard drinks in one sitting, is a particularly concerning feature of this trend [13]. The outcomes of these surveys raise concerns about the immediate health effects of heavy and risky drinking among South African women. The prevalence of anxiety linked to stress and worry was evaluated by several research [13, 26]. Anxiety disease had a lifetime frequency of 5.7–15.8% and a duration of 4.1 to 8.1%, respectively [13, 18]. The point prevalence of generalised anxiety disease was reported in several of these investigations. The high prevalence of generalised anxiety and trauma history in Rwanda during the genocide, according to the authors, indicates that the prevalence of generalised anxiety disease is higher than 10% [13, 27].

Many researchers have observed a marked increase in alcohol consumption throughout the Asian continent over the years, which is a concerning pattern that has been brought to light by the WHO, on Alcohol and Health [28]. Southeast Asia has seen a significant increase in per capita alcohol consumption, with studies showing that between 2000 and 2016, per capita alcohol consumption increased from 2.4 to 4.5 L [28, 29]. Unsettlingly, data from Japan shows that binge drinking is relatively common, with 3.4% of adult females and 12.7% of adult males admitting to partaking in this dangerous habit [28–30]. For decades, the subjects with scientific explanation, psychological research has provided strong predictors of alcohol consumption and anxiety prevalence [31]. According to the European continent, alcohol consumption is a significant public health challenge [32]. Alcohol consumption and alcohol-related diseases are higher in Europe than anywhere else in the world [33, 34].

Moreover, over 60 million people suffer from anxiety disease in a given year. A Eurobarometer survey conducted in June 2023 revealed that 1 in 2 people, about 46% of the European population [18], have experienced anxiety. More than ever, the present experiences emotional or psychosocial problems such as depression or anxiety. This investigation shows that recent rapid technological changes, alcohol distribution and digital marketing have made wine, beer and spirits more popular in Europe [33]. Germany, France and Spain are among the countries with the highest alcohol consumption, which also provides evidence of anxiety prevalence. At the same time, Portugal has the highest anxiety prevalence, with 8,671 cases per 100,000 people [18]. Further, people in Portugal showed a negative correlation between excessive alcohol consumption and significant depression, but a positive correlation between excessive alcohol consumption and anxiety disorders.

One half of the North American population is receiving substance abuse treatment and reporting symptoms, with 50% having high levels of anxiety [26]. In contrast, this finding shows that the general population samples show a significantly lower prevalence of anxiety than alcoholics. Additionally, a severe problem for Canadians is that 72% drink alcohol each year (*WHO*,* 2022)*, showing a significant prevalence of alcohol-related anxiety in Canadians. Thus, the study provides evidence establishing bidirectional causality to support the literature [30, 35]. In the Oceania continent, 94% of the population in countries such as Australia have consumed at least one alcoholic drink in their lifetime. Although the prevalence of anxiety among the population is 6.7%, literature reviewers have pointed out that there is no clear correlation between anxiety from first drunkenness and heavy drinking [36].

This comprehensive literature analysis shows a strong and nuanced correlation between alcohol consumption and the prevalence of anxiety in different geographical areas. Research and theoretical models support the bidirectional nature of this link, which highlights the need for a balanced understanding of the complex interactions between these two components. The significance of fostering mental health, comprehending the worldwide burden of anxiety-related disease, and putting into practice focused therapies that take into consideration the difficulties that every region presents are also emphasised in the review.

This study examines the two-way impact between the prevalence of anxiety and alcohol consumption related to wine, beer, and spirits with a focus on high-income countries using a continental approach. The main goal is to investigate the causal relationship that exists between the prevalence of anxiety and alcohol consumption, making a new contribution to the field in four critical areas.

Primarily, the research encompasses several high-income countries and uses the most recent, comprehensive data gathered over a prolonged duration, a significant divergence from the few studies carried out in this field. Furthermore, the continent-wise analysis improves the knowledge of Granger causality by illuminating the causal relationship and its extent of influence, which are visually depicted by different arrow lengths. This thorough method addresses the need for long-term studies in high-income settings and offers new insights by including a wide range of countries on all six continents.

Finally, the research provides empirical information to support focused strategies aimed at reducing alcohol consumption and addressing mental health issues. The study highlights how this link is changing because of shifting policies and shifting economic situations while also noting the body of research that has already been undertaken on anxiety and alcohol consumption. Thus, the model developed via this study closes a gap yet to be covered in the literature.

## Method

### Study design

The four variables under investigation in this study were anxiety, wine, beer, and spirit, all measured using two different metrics. Anxiety prevalence per 100,000 people served as the metric for anxiety, while the litres of pure alcohol consumed per capita were employed to quantify wine, beer, and spirits, respectively. This analysis focused on the adult population aged 18 and above, providing insights specifically relevant to this demographic. The online resources: WHO and Our World in Data included secondary data used to gather information for this inquiry and the data file used for the study is presented in S1 Appendix. The research covers a total of fifty-two countries, all of which are classified under high-income countries. (One from Africa, nine from Asia, thirty from Europe, six from North America, three from Oceania, and three from South America made up this diverse group of countries.) Over the period from 1990 to 2019, the dataset included annual data for each of the four variables. A solid basis for the study investigation was provided by this dataset, which had 1,560 observations in total. The S2 Appendix in the supplemental materials contains the dataset’s descriptive statistics in full detail.

### Statistical analysis

The methodology of the study is based on Panel Granger causality analysis. This approach applies the Granger causality idea to panel data, which is the collection of data over time on multiple variables. Another level of complexity is introduced by the panel structure, which makes it possible to investigate causal relationships inside and between individual entities and throughout the panel. The Granger causality test is used in panel data settings to determine if the lagged values of one variable reveal meaningful information about another variable for the whole panel. This method considers the dynamic interactions between entities and offers insights into the panel’s aggregate causal relationships across time.

1 $$\:\:\:\:\:\:\sum\:_{K-1}^{P}{\beta\:}_{k}{Y}_{i,t-k}+\sum\:_{K-1}^{P}{\theta\:}_{K}{X}_{i,t-k}+{u}_{i,t}\:\:$$

The independent variable is X, the dependent variable is Y (area and duration are represented by _i_ and _t_, respectively), the error term is u_i, t_, and the frequency of lags is _k_. The distribution of u is normal with u_i, t_ = α_i_ + ε_i, t_; _ρ_ denotes the number of delays, and ε_i, t_ is _i.i.d_. (0, σ_2_). This study’s relationships between anxiety, wine, beer, and spirits make it hard to classify them correctly as dependent and independent variables. For instance, the Granger causality test utilised anxiety as an independent and dependent variable. The study also used the difference of variables to remove lags from the model in Eqs. (2) to (7). The following equations are estimated to test the direction of causality from the anxiety to wine Eq. (2) and from wine to anxiety Eq. (3), anxiety to beer Eq. (4) and from beer to anxiety Eq. (5), anxiety to spirit Eq. (6) and from spirit to anxiety Eq. (7).

2 $$\:{AD}_{i,t}=\sum\:_{k=1}^{\rho\:}\beta\:i{AD}_{i,t-k}+\sum\:_{k=0}^{\rho\:}{\theta\:}_{k}{WC}_{i,t-k}+{q}_{i,t}\:\:$$

3 $$\:{WC}_{i,t}=\sum\:_{k=1}^{\rho\:}\gamma\:i{WC}_{i,t-k}+\sum\:_{k=0}^{\rho\:}{\pi\:}_{k}{AD}_{i,t-k}+{s}_{i,t}\:\:$$

4 $$\:{AD}_{i,t}=\sum\:_{k=1}^{\rho\:}\delta\:i{AD}_{i,t-k}+\sum\:_{k=0}^{\rho\:}{\rho\:}_{k}{BC}_{i,t-k}+{u}_{i,t}\:\:$$

5 $$\:{BC}_{i,t}=\sum\:_{k=1}^{\rho\:}\omega\:i{BC}_{i,t-k}+\sum\:_{k=0}^{\rho\:}{\sigma\:}_{k}{AD}_{i,t-k}+{v}_{i,t}\:\:$$

6 $$\:{AD}_{i,t}=\sum\:_{k=1}^{\rho\:}\vartheta\:i{AD}_{i,t-k}+\sum\:_{k=0}^{\rho\:}{\tau\:}_{k}{SC}_{i,t-k}+{w}_{i,t}\:\:\:$$

7 $$\:{SC}_{i,t}=\sum\:_{k=1}^{\rho\:}\alpha\:i{SC}_{i,t-k}+\sum\:_{k=0}^{\rho\:}{\phi\:}_{k}{AD}_{i,t-k}+{z}_{i,t}\:\:$$

In that order, the prefixes AD, WC, BC, and SC stand for Anxiety Disease, Wine Consumption, Beer Consumption, and Spirit Consumption. The regression coefficients are denoted by the coefficients β_i_, γ_i_, δ_i_, ω_i_, ϑ_i_, and α_i_. For each k in the range [1, N], the constants θ_k_, π_k_, ρ_k_, σ_k_, τ_k_, and φ_k_ stay constant throughout the series. The error terms of the model are denoted by the words Q_i, t_, s_i, t_, u_i, t_, v_i, t_, w_i, t_, and z_i, t_. According to accepted statistical theory, these error factors are assumed to be independent, identically distributed, and have a normal distribution without showing signs of heteroskedasticity or autocorrelation. STATA software was used to do the statistical analysis of the models.

Granger causality analysis is a powerful method of causal analysis that offers several advantages and a deeper understanding. It makes it possible to identify predictive associations, meaning that one variable may predict the values of another in the future, adding directed insights to the investigation. These kinds of revelations can improve the accuracy of forecasts made in the future and deepen our understanding of the dynamic interplay between factors. Furthermore, Granger causality helps identify the main drivers in a dataset by elucidating the lead-lag correlations between variables, which is helpful for academics and policymakers in making strategic decisions. Furthermore, this method provides a solid basis for assessing the causal inferences’ reliability by statistically establishing the strength and importance of these associations. Thus, using Granger causality may significantly improve the breadth and accuracy of causal analyses in social and economic sciences. Further to that as this study focused on short term relationships, we did not conduct a cointegration test. The results presented should therefore be understood within the context of short-term associations rather than long term equilibrium. This method primarily detects linear causal dependencies over time, based on the aggregated nature of the secondary datasets. While this approach offers robust insights into directional causality, it does not inherently account for nonlinear relationships or threshold effects. Future research utilising nonlinear time-series models or machine learning techniques could further explore these dynamics.

## Results

**Fig. 1:**
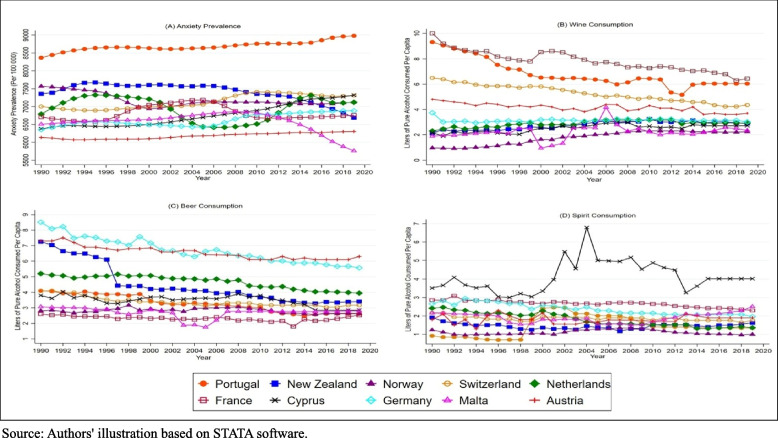
Anxiety prevalence and alcohol consumption patterns. Source: Authors’ illustration based on STATA software

This section initiates significant major discoveries made using the empirical method. First, Fig. 1A − D represents a brief overview of the relevant variables considered, anxiety, wine, beer, and spirits, by revealing their fluctuations in the timeframe. Second, the results of the unit root test. Third, the criteria for determining the lag length and the vector autoregression estimation. Finally, a thorough investigation of the results obtained from the Panel Granger causality test is provided.

Based on Fig. 1A, Portugal has continuously recorded the frequent prevalence of anxiety among the high-income countries shown, with the rate of increase continuing unabated throughout the years. This suggests that mental health trends vary among these countries, with Portugal needing special attention because of its growing trend of anxiety prevalence and Malta exhibiting strong progress in mental health due to its decreasing trend of anxiety prevalence. The greater consistency observed in other countries may point to consistent reporting and diagnostic standards or well-established and functional mental health infrastructures. According to Fig. 1B, Portugal is the biggest consumer of wine among the listed countries, despite a notable decline in per capita consumption since 1990 that has stabilised slightly recently. Despite having a lower baseline, Malta has gradually increased in recent years, either because of tourism or shifting local preferences. In Fig. 1C, Germany tops the group with the highest initial beer consumption, which gradually declines until the early 2000 and then enters a relatively stable phase, with occasional slight fluctuations, maybe due to a mature market or shifting consumer tastes. As shown in Fig. 1D, Cyprus has a dynamic tendency, with a notable peak in spirit consumption in the early 2000s, followed by a steep fall before levelling off to a stable state. The rise and decline in consumption in Cyprus may result from changes in the economy in general. Still, low and stable rates elsewhere point to a shift toward alternative beverages or moderate drinking.

According to the S2 Appendix, Portugal has the highest anxiety prevalence, and Japan has the lowest. Consistent with its renowned vino culture, France leads the way in wine consumption. However, the countries with the lowest wine consumption include Brunei, Oman, and Saudi Arabia. The country with the most beer consumption is Ireland, followed by Saudi Arabia, which has the lowest due to stringent alcohol laws imposed for religious reasons. The Bahamas is the most significant country with the most consumption of spirits, while Saudi Arabia has the lowest. Finally, a notable trend in the consumption of wine, beer, and spirits is shown, and Saudi Arabia is found to have the lowest recorded amounts in each category. The significance of strict alcohol legislation and cultural norms on Saudi Arabia’s broad patterns of alcohol consumption are highlighted by this consistency. The information shows that different kinds of alcoholic beverages in the nation adhere to these regulations differently.

### Unit root test

Preceding the Granger causality test, the researchers investigated the stationarity of anxiety in connection to wine, beer, and spirits, respectively. The study served the Dickey-Fuller unit root test, developed by David Dickey and Wayne Fuller in 1979. This test’s null hypothesis is that there is a unit root, which implies the data arrangement is not stationary. The alternative hypothesis is, as a rule, stationarity or trend stationarity but may shift depending on the adaptation of the test utilised.

In statistics, a unit root test tests whether a variable is non-stationary, utilising an autoregressive demonstration. The Dickey-Fuller unit root test is a fairly well-known test in massive tests. The analysts tested for non-stationarity within the conveyance of anxiety prevalence, wine, beer, and spirits pure alcohol litres per capita before the Granger causality examination. Mostly, anxiety remained stable when tested under the third difference (dddAnxiety) and similar to this, the three alcohol consumption patterns; wine, beer, and spirits showed stable when tested under the first difference (dWine, dBeer, and dSpirit) of this study.

The S3 Appendix reports the results of the time-series unit root test proposed by Dickey and Fuller for the anxiety prevalence per 100,000 people and alcohol consumption patterns: wine, beer, and spirits litres of pure alcohol consumed per capita.

### Lag length criteria

In panel data analysis, choosing an optimal lag length is critical to building robust models that accurately capture the underlying causal relationships in the data. A selection of lag length criteria guides this method, each offering a unique perspective on the trade-off between complexity and model fit. A number of important criteria are useful in selecting the proper lag order: Firstly, a statistical technique for evaluating model accuracy is provided by the Akaike Information Criterion. Secondly, Schwarz’s Bayesian Information Criterion is used to estimate the accuracy of model complexity. Thirdly, a compromise between model fit and simplicity is offered by the Hannan-Quinn Information Criterion. Finally, evaluating the model’s forecast error requires consideration of the Final Prediction Error Criteria. When combined, these methods help achieve the best possible lag order selection. Together, these criteria provide a multifaceted lens through which to navigate the complex landscape of regression analysis, ensuring a well-informed choice aligned with the characteristics and goals of the panel data under examination.

### Vector autoregression estimation

The Granger causality test relies heavily on this assumption, which offers a thorough framework for examining the temporal relationships between the four variables: anxiety, wine, beer, and spirits. Vector autoregression models represent the dynamic interactions between various time-series variables in the setting of Granger causality. Considering the lagged values of the variables, the Vector autoregression estimate permits the concurrent investigation of the causal relationships between the variables. This approach makes it possible to determine if the historical values of one variable include information that aids in predicting the current values of another by calculating the coefficients of the lag variables. A robust statistical method for investigating the temporal precedence and directionality of causal relationships among variables throughout time is offered by Vector autoregression estimates in Granger causality testing.

### Granger causality test

A statistical technique called Granger causality is employed to evaluate the causal relationship between two time-series data. This method, which bears Clive Granger’s name, goes beyond conventional correlation analysis by investigating if information from one variable’s historical values may be used to forecast the future values of another. The Granger causality test is based on the idea that if the first variable is a cause of the second variable, then previous values of the first variable should provide information that may be used to forecast future values of the second variable. There are many procedures involved in doing a Granger causality test. To decide how many historical observations to consider, the first step is to choose the pertinent time-series variables and define the lag order. This is essential since it affects the identification of the causal relationship between variables.

The next step is to estimate a Vector autoregression model after establishing the variables and lag order. Examining the variables’ interdependencies across time is made possible by the Vector autoregression model, which represents the joint evolution of the selected variables. The relevance of the lagged values of one variable in forecasting the future values of another is evaluated using statistical tests after the estimation of the Vector autoregression model. In a Granger causality test, the null hypothesis states that previous values of the first variable do not contribute to the second variable’s prediction beyond the earlier values of the first variable. Indicating that the first variable does have a predictive impact on the second, the rejection of this null hypothesis suggests the existence of Granger causality. It is crucial to understand that Granger causality finds predicted associations based on temporal patterns rather than establishing actual causation in the conventional sense. The context and confounding variables affecting the identified associations should be carefully evaluated when assessing the results. The Granger causality approach entails choosing variables, defining lag orders, estimating a Vector autoregression model, and running statistical tests to determine the relevance of predicting correlations between the selected variables across time. For further details on the Granger causality methodology, these articles would provide a comprehensive reference [15, 37, 38].

A novel analytic model was constructed for this study under the Granger causality test to describe the interrelationships between each category and depict the data produced by the causal relationship between anxiety across wine, beer, and spirits; the investigation of causal relationships between anxiety and the consumption of wine, beer, and spirits was analysed separately under the Granger causality test. Anxiety about wine, anxiety about beer, and anxiety about spirit were all subjected to this analytical procedure separately for each pair, with different outcomes for each interaction. The results of these studies were thoroughly collated, and the correlation between the variables was visually shown with arrows. Interestingly, these arrows help to indicate the causal relationship and the degree of influence.

Consequently, the lengths of the arrows were allocated variably to improve the graphical representation and highlight the strength of the established causal relationships by verifying the stationarity of the variables and the differences that pro for each variable in this study four different arrow lengths; very small, small, medium and large specifically used. In addition to enabling a more nuanced representation of the causal relationships, this intentional variation in arrow lengths also offers a visual representation of the relative weights that each variable has on the others. The highest degree of influence between the two variables under consideration by the tiny arrow. A proportionate decrease in the degree of influence between the variable pairs is observed when the arrow length grows from small to large. This deliberate variation in arrow lengths is a visual cue highlighting the complex strength of the causal relationships and offers. It offers a simple and understandable way to comprehend the relative effect of each variable.

Table 1 summarises high-income countries’ analysis of anxiety about wine, beer, and spirit from the Granger causality test under a continental analysis. In the Granger causality study, Seychelles stands out among African countries, showing a one-way causal relationship from anxiety to wine, beer, and spirit consumption. This implies that anxiety to alcohol consumption has a one-way right-directional causal relationship in the Seychelles in all three categories.

**Table 1:**
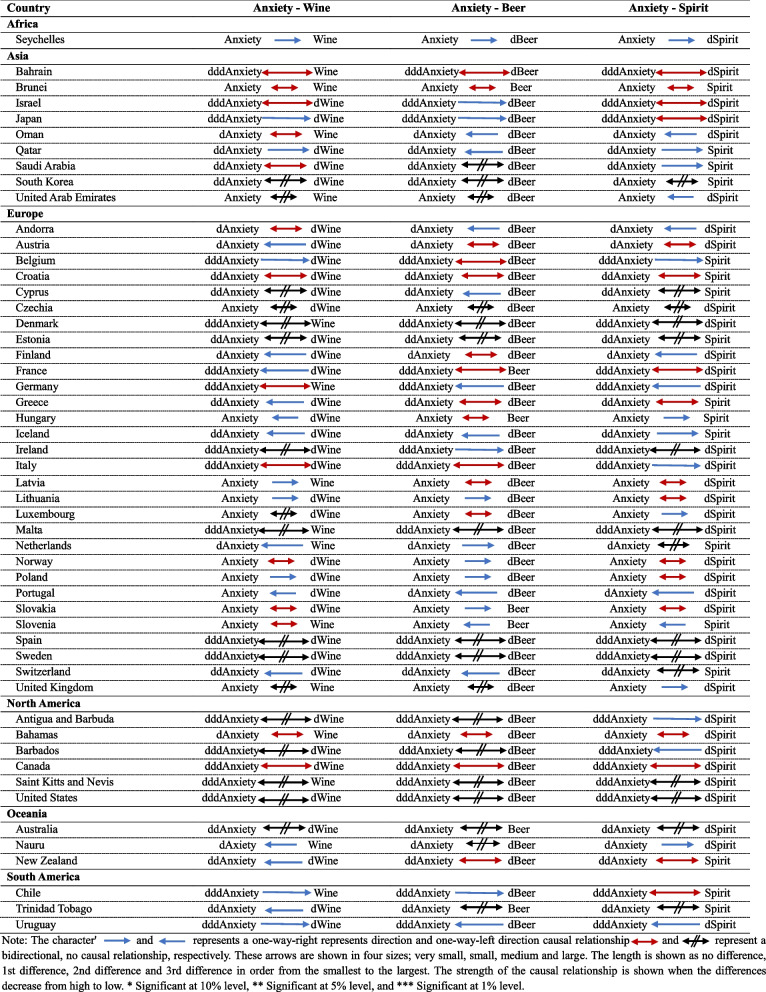
High-income countries analysis for anxiety from Panel Granger causality

Examining the Asian continent in more detail, Japan and Qatar show a one-way right-direction causal relationship among nine high-income countries between anxiety and wine consumption. Conversely, a bidirectional relationship is seen in Bahrain, Brunei, Israel, Oman, and Saudi Arabia, highlighting a reciprocal impact between anxiety and wine consumption. However, there is no evidence of a clear causal relationship between anxiety and wine consumption in South Korea or the United Arab Emirates. Regarding anxiety about beer consumption, a one-way right-direction causal relationship is represented by Israel and Japan. In contrast, a one-way left-direction causal relationship is represented by Oman and Qatar. Further, a bidirectional causal relationship is demonstrated by Bahrain and Brunei. Finally, no causal relationship exists between Saudi Arabia, South Korea, and the United Arab Emirates. There is a bidirectional causal relationship between anxiety and spirit consumption in Bahrain, Brunei, Israel and Japan. In contrast, a one-way right-direction causative relationship is evident in Qatar and Saudi Arabia, and a one-way left-direction causative relationship is represented in Oman and the United Arab Emirates. Lastly, South Korea does not demonstrate a causal relationship between the two variables on the Asian continent.

To summarise, Bahrain and Brunei exhibit a bidirectional causal relationship in all three categories of alcohol consumption within the Asian continent. Between those two countries, Brunei stands out with the most significant influence on the bidirectional causal relationship that links anxiety and wine, beer, and spirit consumption. The three Asian consumption trends have no causal relationship with South Korea.

For the European continent, nine countries show a one-way left-direction causal relationship from wine consumption to anxiety prevalence, and four countries out of all thirty high-income countries show a one-way right-direction causative relationship from anxiety to wine consumption. Andorra, Croatia, Germany, Italy, Norway, Slovakia, and Slovenia display bidirectional causality. There was no Granger causal relationship between anxiety and wine consumption in ten high-income countries. Additionally, seven countries showed a one-way left causality from beer consumption to anxiety, and six countries showed a proper one-way right causality from anxiety to beer consumption. Ten countries out of all thirty high-income countries in Europe observed bidirectional Granger causality for anxiety and beer consumption. There was no causal relationship between anxiety and beer consumption in the remaining countries; Czechia, Denmark, Estonia, Malta, Spain, Sweden and the United Kingdom. Regarding anxiety and spirit consumption, observations showed a one-way right direction causal relationship anxiety to spirit consumption among Belgium, Italy, Iceland, Ireland, Luxembourg, and the United Kingdom. In contrast, five countries opposed a one-way left-directional Granger causality from spirit consumption to anxiety. A bidirectional Granger causality was observed in nine countries. Cyprus, the Czechia, Denmark, Estonia, Ireland, Malta, Netherlands, Spain, Sweden, and Switzerland are observed to show that spirit consumption does not cause anxiety, and anxiety does not cause spirit consumption.

Moreover, in the European continent, only Croatia exhibits a bidirectional causal relationship in all three categories of alcohol consumption with anxiety. In addition, only Portugal reaches a one-way left-direction causal relationship for all three consumption categories of wine, beer, and spirit to anxiety. The countries of Czechia, Denmark, Estonia, Malta, Spain, and Sweden do not show any Granger causal relationship between the prevalence of anxiety and all three categories of alcohol consumption.

North America, the Bahamas and Canada showed a bidirectional causal relationship between anxiety and wine consumption in the data set. Besides, four out of all six countries observed no causality between anxiety and wine consumption. In the North American continent, the results shown by anxiety and beer consumption are like the above results, which are related to anxiety and wine consumption in the same continent. However, the relationship between spirit consumption and anxiety is different. The Bahamas and Canada showed bidirectional Granger causality. A one-way right-directional Granger causality from anxiety to spirit consumption was observed only in Antigua and Barbuda. A one-way left-directional Granger causality from spirit consumption to anxiety was represented only in Barbados. Saint Kitts and Nevis and the United States showed no causal relationship between spirit consumption and anxiety.

In summary, in the North American continent, only the Bahamas and Canada show a bidirectional causal relationship for all three categories of alcohol consumption with anxiety prevalence. Saint Kitts and Nevis and the United States showed no causal relationship between wine, beer, spirit consumption and anxiety. Notably, several of the same countries do not exhibit a unidirectional relationship with anxiety in all three alcohol consumption categories simultaneously.

The relationship between anxiety prevalence and types of alcohol consumption separately in Oceania’s countries, both Nauru and New Zealand, showed a one-way left-direction causality from wine consumption to anxiety. For this continent, bidirectional causality between anxiety and wine consumption was not observed in any country. There was no significant causal relationship observed between anxiety and wine consumption in Australia. As well, only New Zealand showed a bidirectional relationship between beer consumption and anxiety. No causal relationship was demonstrated between beer consumption and anxiety prevalence between Australia and Nauru. Additionally, Australia showed no causal relationship between spirit consumption and anxiety prevalence. In contrast, only Nauru showed a one-way right-direction Granger causality from anxiety prevalence to spirit consumption, while only New Zealand showed bidirectional causality.

Considering all three categories of alcohol consumption in Oceania in general, only New Zealand shows a bidirectional relationship between beer and spirit consumption and anxiety. Furthermore, anxiety does not show any relationship with wine, beer, and spirit consumption, respectively, in Australia.

Among high-income countries in South America, Tobago observed a one-way left-direction Granger causality from wine consumption to anxiety. In contrast, Chile and Uruguay showed a one-way right-direction Granger causality from anxiety prevalence to wine consumption. Anxiety about beer consumption, Chile showed a one-way right-directional relationship from anxiety to wine consumption, Trinidad and Tobago do not show any causal relationship between these two variables and Uruguay observed a one-way left-directional relationship from beer consumption to anxiety. Anxiety about spirit consumption, Chile, Trinidad and Tobago, and Uruguay showed bidirectional, no causality, and a one-way left-directional relationship, respectively.

When comparing the results of South America in general, Only Chile showed a bidirectional relationship between anxiety and spirit consumption. Uruguay, Trinidad and Tobago, and Chile showed a unidirectional relationship only for wine consumption from all three categories. In Trinidad and Tobago; beer and spirits, do not show any causality relationship with anxiety.

S4 Appendix shows the fifty-two high-income countries analysis for anxiety prevalence and wine consumption from Panel Granger causality under a continental analysis. S5 Appendix presents the fifty-two high-income countries analysis for anxiety prevalence and beer consumption from Panel Granger causality under a continental analysis. S6 Appendix observes the fifty-two high-income countries analysis for anxiety prevalence and spirit consumption from Panel Granger causality under a continental analysis. These Granger causality test results reveals that there are diverse patterns in the causal relationship between anxiety and the consumption of wine, beer, and spirits varies throughout African, Asian, European, North American, Oceanian, and South American continents.

## Discussion

The primary focus of this study was to determine the causality of wine, beer, and spirit consumption for anxiety. According to the findings of the study, the relationship between alcohol consumption and anxiety prevalence varies across different continents over time. In the African continent, Seychelles stands out for demonstrating a one-way causal association between anxiety and alcohol consumption. Bahrain and Brunei demonstrate bidirectional causality in all three categories of alcohol consumption within the Asian continent. Brunei has the most significant effect on bidirectional causal relationships linking anxiety to wine, beer, and spirits consumption. In line with the WHO, compared to other regions, there is a markedly higher incidence of anxiety disorders in Southeast Asia [35]. Moreover, Brunei is a country in Southeast Asia, and the impact on anxiety is evident. However, no causal relationship between wine, beer, spirit consumption, and anxiety were found in South Korea.

However, it is important to clarify that this does not directly examine the effects of shifting policies or economic situations on these relationships. While these factors may contribute to the observed trends, our results do not provide empirical evidence supporting this claim. The finding indicates varying patterns of causation across regions and while we acknowledge that shifting public health policies and economic conditions may influence these dynamics and further research is necessary to explore these dynamics. Alcohol-related consequences were often associated with anxiety, regardless of gender [39]. The results support the existing literature, concluding that responses from different parties confirm a causal relationship [30]. In summary, among all high-income countries in Europe, only Croatia shows a bidirectional relationship between anxiety and consumption of wine, beer, and spirits. People with anxiety disorders are more likely to become dependent on alcohol because they use it to self-medicate or view these people’s anxiety disorders as an artefact that can be primarily relieved by alcohol [14]. Furthermore, in Europe, the Czechia Republic is the only country that has no relationship between alcohol consumption and anxiety. Portugal shows a unidirectional relationship between anxiety for all three consumptions of wine, beer, and spirits. For instance, Portugal has the highest anxiety prevalence rate among high-income European countries [40]. Recent studies have shown a high prevalence of anxiety in the elderly population of Portugal, which is a matter of concern [18, 40]. The least influential countries for wine, beer, and spirits in Europe are Denmark, Malta, Sweden, and Spain. When beer, wine, and spirits consumption were analysed simultaneously among those countries, a lower risk was observed for wine consumers in Sweden [33].

While this study provides valuable insights into the relationship between alcohol consumption and anxiety between various continents, it is essential to consider diverse socio-cultural contexts that may influence these dynamics particularly within the high-income countries. For example, societal attitudes towards alcohol and mental health can vary significantly affecting how individuals perceive and respond to anxiety and their alcohol consumption habits. For instance, in some cultures, alcohol use may be deeply ingrained in social traditions, which can normalise consumption even in situations where individuals are experiencing anxiety. Conversely, in societies where alcohol use is heavily stigmatised or regulated, individuals might adopt different coping mechanisms, potentially obscuring the relationship between alcohol consumption and anxiety disorders.

Economic disparities also play a critical role in shaping this relationship. High-income countries often have greater access to mental health services and public health resources, which can mediate the impact of anxiety disorders and alcohol use. However, even within these countries, disparities in healthcare accessibility and affordability can create unequal outcomes.

Cultural stigma associated with mental health issues is another factor that varies widely across countries. In some societies, the stigma may discourage individuals from seeking help for anxiety or related disorders, potentially leading to self-medication through alcohol. Additionally, differences in public health policies, availability of alcohol, and legal regulations regarding its consumption further contribute to the variation in how this relationship manifests across nations.

By integrating these socioeconomic and cultural factors into the discussion, we emphasize the need for a more nuanced understanding of the relationship between alcohol consumption and anxiety disorders. This perspective is essential for designing targeted interventions and public health policies that are culturally and contextually appropriate.

Additionally, a notable limitation of this study is the lack of detailed demographic data, such as age and gender, within the secondary datasets utilised (sourced from WHO and Our World in Data). This limitation restricts our ability to conduct a granular analysis of the relationship between alcohol consumption and anxiety disorders across specific demographic groups. Understanding such variations is crucial for developing a nuanced understanding of how these factors interplay. Future research should prioritise the collection and analysis of age- and gender-specific data to provide deeper insights into the demographic dynamics influencing this relationship. Such studies will enable a more tailored approach to addressing public health concerns.

Furthermore, the findings, which found no causal relationship between anxiety and the type of alcohol in the United States, are also substantially different to the literature [21, 41]. The differences in findings in the literature could be attributed to various factors, such as these findings may relate to many factors contribute to these findings, including social isolation, social support, and differences in financial stability [41, 42]. For those reasons, unmarried adults and young adults in the United States are showing the fastest increase in anxiety [41]. Similarly, a unidirectional causal relationship was detected between anxiety and consumption of wine, beer, and spirits. For instance, our results differ from the literature in that 6.7% of the general population in Australia have a first-onset anxiety disorder, which increases significantly with first-onset al.cohol consumption [36].

On the one hand, mental health problems such as anxiety are more likely to be self-medicated through readily available substances such as wine, beer, and spirits. However, self-reported anxiety rates in heavy drinkers may not accurately reflect actual anxiety levels. Their symptoms can be wholly or partially relieved through alcohol [18].

In the North American continent, only Canada and the Bahamas showed a bidirectional relationship with wine, beer, and spirit consumption that increased the risk of anxiety disorders with heavy alcohol consumption [14, 26]. Anxiety was negatively associated with general alcohol use, and positive, parallel associations were found between anxiety and heavy alcohol consumption, demonstrating that anxiety is the most common condition analysed in Canada and affects approximately 10% of people [43].

While considering differences in wine, beer, and spirit consumption among high-income countries in the two continents of Oceania and South America, the bidirectional relationship is only shown for beer and spirits in New Zealand. In Oceania, Australia’s anxiety prevalence has no causal relationship with alcohol. Uruguay is the only country in South America that has one-way relations. Anxiety is common in Latin America. Estimates in this area are similar to high-income European countries in the literature [31]. A Granger causality study looked at the relationship between anxiety and wine, beer, and spirit consumption, respectively, where high-income earners were a risk factor for anxiety due to alcohol consumption. Therefore, the insights provided by this study will be necessary for health.

Furthermore, social cultural factors such as stigma surrounding mental health, access to treatments, and community support can also mediate the interplay between alcohol consumption and anxiety. For instance, in nations with strong social support networks, individuals may be less likely to resort alcohol as a coping mechanism while those in less supportive environments may turn in to drinking as a means of managing their anxiety. The findings of this study highlight the necessity of considering these diverse social cultural contexts when interpreting the relationship between alcohol consumption and anxiety. Further research should aim to incorporate these factors to gain a more comprehensive understanding of how social cultural dynamics influence drinking behaviors and mental health outcomes across different high-income settings.

### Contribution of the study

This work has a broad and substantial impact on the fields of behavioral economics and public health. Granger causality analysis is used to examine the dynamic links between the prevalence of anxiety and alcohol consumption, including wine, beer and spirits across a number of high-income countries. The study reveals a variety of patterns of causation using a careful research design and a thorough statistical analysis. It presents bidirectional relationships in certain continents, one-way relationships in others, and no causal relationships in a few cases.

The research is noteworthy because it takes a continental approach, delivering insightful information on how various socioeconomic and cultural factors impact these linkages differently depending on the region. The study’s creative methodology also incorporates a brand-new graphical depiction that improves interpretability by graphically illustrating the strength of confirmed causal linkages using arrows of different lengths.

Crucially, by undertaking a thorough analysis of these relationships over a prolonged period of time, the study closes an empirical vacuum that had not been adequately addressed by earlier studies in high income countries. The research greatly aids in the development of focused therapies and policymaking by determining the direction and strength of the relationship between alcohol consumption and anxiety.

As a result, taking into account the Granger causality approach, the research broadens the scope of scholarly writing and may help guide the development of policies that enhance public health and social well-being.

### Policy implications

This study highlights the intricacy of the link between anxiety and alcohol consumption, which is impacted by a wide range of variables such as public health policy, cultural norms, and economic position. The different results for different alcohol patterns and regions highlight the need for more studies to uncover the underlying mechanisms of these relationships and for customised public health initiatives. This work lays the groundwork for future research and policymaking in different countries by advancing the understanding of mental health and alcohol consumption.

Globally, as the prevalence of anxiety disorders increases in high-income countries, as alcohol use increases, policy implementation is preferable. Policy making needs to be carefully managed. The findings of this study suggest that anxiety disorder has both positive and negative effects. This phenomenon provides valuable policy implications for relevant governments aiming to reduce anxiety and alcohol consumption [44]. Among high-income countries, meaningful and sustained awareness of alcohol-related diseases is needed to reduce the harm caused by excessive alcohol consumption, focusing on countries with high populations, particularly on the European continent [45]. A higher level of alcohol control policy activity is expected as a result. A few countries should ban alcohol to the purchase or consumption of alcohol [45]. It is fitting to develop a national written alcohol policy and awareness activities.

As a third policy change with high-income demographics, government health objectives ought to incorporate the improvement of programmes that recognise and support individuals at risk for anxiety disorders at an early arrange, preventive measures, and early mediation [44]. Moreover, there is a constant need for changes and new approaches. The WHO should propose creating a local tax system in its member countries, and the presence of the illegal market for alcohol can be controlled through policy measures such as charge arrangements in numerous nations [46]. Decreasing the mental pressure of a country’s workforce will expand the workers’ most economic efficiency. Further to the above policies, to effectively address the intersection of alcohol consumption and anxiety disorders, it is imperative for policymakers to account for demographic diversity, particularly age and gender differences. While our findings underscore the significance of this relationship, the absence of granular demographic data highlights the need for interventions to be guided by comprehensive datasets that capture these variations. Policymakers should prioritise initiatives that integrate detailed demographic analyses to design more inclusive and targeted prevention and treatment strategies.

Fourth, increasing the price of alcoholic beverages through taxation and other pricing policies, reducing the availability of alcoholic beverages and banning the advertising and sale of alcoholic beverages. For example, in Lithuania, in 2018, off-premises purchase of alcoholic beverages is restricted to Monday through Saturday from 10 a.m. to 8 p.m. and from 10 a.m. to 3 p.m [32, 45]. Imposing such regulations requires cooperation among governments, healthcare systems, educational institutions, and advisory groups to implement and enforce these regulations globally. It is an ongoing process that involves constant evaluation and adaptation to meet the evolving needs of people worldwide. Finaly it is crucial to nuance the fact that our study employed Panel Granger Causality Analysis, which focuses on linear temporal relationships between the variables. While this approach is robust for identifying directional causality, it does not capture potential nonlinear interactions or threshold effects. Factors such as policy shifts, economic crises, or cultural events may introduce nonlinear dynamics into these relationships. Future research employing nonlinear methodologies, such as regime-switching models or machine learning techniques, could offer a deeper understanding of these complexities.

## Conclusion

The complex relationships between anxiety and alcohol consumption across continents in high-income countries are shown by this extensive study. The results demonstrate a variety of patterns of causation, with some areas demonstrating a bidirectional relationship where anxiety drives alcohol use and others demonstrating a one-way relationship. Yet, others having no discernible causal association. Interestingly, unique causal relationships across wine, beer, and spirits are shown in all countries.

In African countries, Seychelles stands out for demonstrating a one-way causal relationship between anxiety and alcohol consumption. The different degrees of one-way and bidirectional causation across Asia, especially in Bahrain, Brunei, and Japan, which are high income earning countries highlight the socioeconomic and cultural influences on these dynamics. In all three alcohol categories, Croatia has the strongest bidirectional association among the European nations, which exhibit a combination of unidirectional and bidirectional causation. The picture in North America is more uniform, with the Bahamas and Canada exhibiting bidirectional causation for all forms of alcohol. In contrast, no causal association was seen in the United States or Saint Kitts and Nevis. With countries like New Zealand and Uruguay exhibiting their tendencies, Oceania and South America demonstrate the significance of regional context in comprehending the anxiety-alcohol consumption nexus. The lack of a link in several nations and categories suggests that social, legal, or cultural factors may moderate these correlations.

The results of this study might help international organisations and decision-makers, such as governments, understand the actual effects of the past three decades’ worth of policy execution on anxiety prevalence and alcohol consumption. These authorities and players can create more successful policies in the future to accomplish their desired goals by drawing on the lessons acquired from prior execution. This research study makes it easier to conduct a more thorough analysis of the characteristics of anxiety prevalence and alcohol consumption in various countries. Using the same data set, a potential expansion of this research would look at the nature and causation of the relationship between the prevalence of anxiety and tobacco use by country. Furthermore, moderator factors such as socioeconomic level under gross domestic product, which may have a significant impact on the relationship between anxiety prevalence and tobacco use, might add more value to a future study.

## Supplementary Information

Additional file 1. S1 Appendix. Data file

Additional file 2. S2 Appendix. Descriptive statistics for the data set

Additional file 3. S3 Appendix. LLC unit root test

Additional file 4. S4 Appendix. High-income countries analysis for anxiety and wine from Panel Granger causality

Additional file 5. S5 Appendix. High-income countries analysis for anxiety and beer from Panel Granger causality

Additional file 6. S6 Appendix. High-income countries analysis for anxiety and spirits from Panel Granger causality

## Data Availability

All data generated or analysed during this study are included in this published article and its supplementary information files.
