# Supplementary material for: Evaluating the synergy: anxiety prevalence and alcohol consumption patterns in high-income countries using Granger causality analysis
Source: BMC Public Health. 2025 Jan 20;25:220. doi: 10.1186/s12889-025-21402-6 (PMC11744946; doi:10.1186/s12889-025-21402-6)
Supplement: Supplementary file 2 — Additional file 2. S2 Appendix. Descriptive statistics for the data set [file 12889_2025_21402_MOESM2_ESM.docx]

**S2 Appendix. Descriptive statistics for the dataset.**

| **Country** | **Type** | **Mean** | **Std. dev** | **Min** | **Max** |
| --- | --- | --- | --- | --- | --- |
| **Andorra** | Anxiety | 6017.32 | 330.01 | 5400.18 | 6706.55 |
|  | Wine | 6.26 | 1.52 | 4.13 | 9.03 |
|  | Beer | 3.22 | 0.12 | 2.96 | 3.59 |
|  | Spirit | 2.91 | 0.47 | 2.32 | 3.70 |
| **Antigua and Barbuda** | Anxiety | 4425.62 | 212.73 | 4003.41 | 4617.54 |
|  | Wine | 1.68 | 1.00 | 0.51 | 3.95 |
|  | Beer | 2.07 | 0.75 | 0.64 | 2.97 |
|  | Spirit | 3.34 | 0.82 | 1.27 | 4.55 |
| **Australia** | Anxiety | 5664.46 | 150.57 | 5377.05 | 5967.37 |
|  | Wine | 3.29 | 0.45 | 2.66 | 3.87 |
|  | Beer | 4.81 | 0.84 | 3.68 | 7.08 |
|  | Spirit | 1.44 | 0.29 | 1.12 | 2.07 |
| **Austria** | Anxiety | 6176.74 | 80.27 | 6069.20 | 6303.34 |
|  | Wine | 4.14 | 0.32 | 3.6 | 4.8 |
|  | Beer | 6.56 | 0.41 | 6.1 | 7.5 |
|  | Spirit | 1.81 | 0.21 | 1.3 | 2.3 |
| **Bahamas** | Anxiety | 4143.20 | 145.74 | 3950.94 | 4396.10 |
|  | Wine | 1.39 | 0.23 | 0.81 | 1.98 |
|  | Beer | 2.98 | 1.07 | 0.85 | 4.03 |
|  | Spirit | 5.71 | 2.10 | 3.69 | 9.84 |
| **Bahrain** | Anxiety | 5060.26 | 325.38 | 4652.89 | 5678.11 |
|  | Wine | 0.12 | 0.03 | 0.09 | 0.24 |
|  | Beer | 0.76 | 0.50 | 0.34 | 2.26 |
|  | Spirit | 1.81 | 1.06 | 0.66 | 3.99 |
| **Barbados** | Anxiety | 4579.35 | 282.43 | 4284.35 | 5078.28 |
|  | Wine | 0.77 | 0.43 | 0.25 | 1.78 |
|  | Beer | 2.94 | 0.56 | 1.83 | 3.66 |
|  | Spirit | 4.12 | 0.40 | 3.25 | 4.8 |
| **Belgium** | Anxiety | 5189.27 | 70.75 | 5081.18 | 5279.70 |
|  | Wine | 3.64 | 0.44 | 2.59 | 4.36 |
|  | Beer | 5.60 | 0.86 | 4.35 | 4.37 |
|  | Spirit | 1.44 | 0.53 | 0.92 | 3.96 |
| **Brunei** | Anxiety | 2923.95 | 96.67 | 2735.01 | 3029.05 |
|  | Wine | 0.02 | 0.02 | 0.00 | 0.10 |
|  | Beer | 0.60 | 0.66 | 0.08 | 2.42 |
|  | Spirit | 0.13 | 0.15 | 0.01 | 0.49 |
| **Canada** | Anxiety | 4353.31 | 435.69 | 3666.34 | 4769.12 |
|  | Wine | 1.57 | 0.38 | 1.1 | 2.1 |
|  | Beer | 4.14 | 0.29 | 3.5 | 4.71 |
|  | Spirit | 2.08 | 0.18 | 1.9 | 2.74 |
| **Chile** | Anxiety | 5582.97 | 184.40 | 5332.64 | 5810.87 |
|  | Wine | 2.77 | 0.50 | 2.02 | 4.37 |
|  | Beer | 2.21 | 0.40 | 1.6 | 2.85 |
|  | Spirit | 2.42 | 0.32 | 1.9 | 3.15 |
| **Croatia** | Anxiety | 3999.71 | 105.41 | 3854.19 | 4235.19 |
|  | Wine | 5.20 | 1.10 | 3.52 | 7.6 |
|  | Beer | 4.52 | 0.53 | 3.17 | 5.26 |
|  | Spirit | 1.73 | 0.80 | 1.02 | 4.4 |
| **Cyprus** | Anxiety | 6753.08 | 311.44 | 6378.43 | 7318.93 |
|  | Wine | 2.50 | .29 | 1.89 | 2.96 |
|  | Beer | 3.47 | .33 | 2.85 | 4.03 |
|  | Spirit | 4.13 | .85 | 3 | 6.78 |
| **Czechia** | Anxiety | 3562.61 | 49.56 | 3446.92 | 3.618.59 |
|  | Wine | 2.42 | .24 | 2.11 | 2.77 |
|  | Beer | 7.30 | .44 | 6.64 | 8.22 |
|  | Spirit | 3.66 | .35 | 3.04 | 4.16 |
| **Denmark** | Anxiety | 5040.04 | 52.83 | 4980.68 | 5125.74 |
|  | Wine | 4.10 | .52 | 2.82 | 5.04 |
|  | Beer | 5.22 | 1.50 | 3.42 | 7.45 |
|  | Spirit | 1.52 | .15 | 1.21 | 1.84 |
| **Estonia** | Anxiety | 3744.80 | 66.56 | 3606.23 | 3822.34 |
|  | Wine | 1.27 | .67 | .1079 | 2.35 |
|  | Beer | 4.45 | 1.61 | 1.67 | 6.5 |
|  | Spirit | 5.72 | 1.37 | 3.21 | 8.36 |
| **Finland** | Anxiety | 3941.83 | 225.78 | 3664.47 | 4365.54 |
|  | Wine | 1.56 | .27 | 1.02 | 1.91 |
|  | Beer | 4.42 | .26 | 3.76 | 4.89 |
|  | Spirit | 1.63 | .58 | 1.07 | 2.86 |
| **France** | Anxiety | 6816.93 | 200.29 | 6575.85 | 7188.58 |
|  | Wine | 7.83 | .84 | 6.29 | 10.01 |
|  | Beer | 2.31 | .16 | 1.8 | 2.6 |
|  | Spirit | 2.66 | .17 | 2.3 | 3.09 |
| **Germany** | Anxiety | 6617.82 | 171.10 | 6355.05 | 6882.26 |
|  | Wine | 3.13 | .13 | 2.93 | 3.73 |
|  | Beer | 6.69 | .81 | 5.57 | 8.51 |
|  | Spirit | 2.37 | .29 | 1.97 | 2.95 |
| **Greece** | Anxiety | 5978.98 | 22.26 | 5925.56 | 6003.98 |
|  | Wine | 3.89 | .60 | 2.66 | 4.71 |
|  | Beer | 2.29 | .14 | 2.05 | 2.55 |
|  | Spirit | 2.28 | .63 | 1.45 | 3.34 |
| **Hungary** | Anxiety | 3864.84 | 52.80 | 3762.75 | 3925.53 |
|  | Wine | 3.97 | .62 | 2.93 | 4.88 |
|  | Beer | 4.43 | .68 | 3.78 | 6.59 |
|  | Spirit | 4.03 | 0.41 | 3.5 | 5.39 |
| **Iceland** | Anxiety | 5206.46 | 45.87 | 5070.85 | 5275.95 |
|  | Wine | 1.57 | .52 | .76 | 2.15 |
|  | Beer | 3.19 | .94 | 1.45 | 4.42 |
|  | Spirit | 1.61 | .46 | 1.1 | 2.76 |
| **Ireland** | Anxiety | 7013.15 | 293.46 | 6690.47 | 7503.02 |
|  | Wine | 2.09 | .84 | .71 | 3.03 |
|  | Beer | 7.09 | 1.75 | 4.92 | 9.96 |
|  | Spirit | 2.36 | .288 | 1.92 | 3.10 |
| **Israel** | Anxiety | 5698.26 | 336.28 | 5266.50 | 6442.90 |
|  | Wine | 6.06 | 1.20 | 4.45 | 8.22 |
|  | Beer | 1.66 | 0.14 | 1.41 | 1.99 |
|  | Spirit | 0.90 | 0.17 | 0.58 | 1.19 |
| **Italy** | Anxiety | 5698.26 | 336.28 | 5266.50 | 6442.90 |
|  | Wine | 6.06 | 1.20 | 4.45 | 8.22 |
|  | Beer | 1.66 | .14 | 1.41 | 1.99 |
|  | Spirit | .90 | .17 | .58 | 1.19 |
| **Japan** | Anxiety | 2756.55 | 169.77 | 2474.75 | 2904.20 |
|  | Wine | .28 | .08 | .14 | .4 |
|  | Beer | 2.15 | .77 | 1.35 | 3.55 |
|  | Spirit | 2.33 | .91 | .74 | 3.411 |
| **Latvia** | Anxiety | 4167.13 | 117.04 | 3942.44 | 4302.20 |
|  | Wine | 1.26 | 0.24 | 0.84 | 1.78 |
|  | Beer | 3.24 | 1.52 | 1.01 | 5.3 |
|  | Spirit | 4.63 | 1.03 | 3.04 | 6.68 |
| **Lithuania** | Anxiety | 4654.48 | 152.92 | 4361.76 | 4801.62 |
|  | Wine | 1.18 | 0.59 | 0.25 | 2.37 |
|  | Beer | 4.13 | 1.29 | 2.08 | 5.79 |
|  | Spirit | 4.23 | 1.39 | 1.16 | 5.81 |
| **Luxembourg** | Anxiety | 5535.81 | 20.77 | 5495.81 | 5603.82 |
|  | Wine | 5.47 | 0.42 | 4.17 | 6.71 |
|  | Beer | 4.50 | 0.46 | 3.84 | 5.55 |
|  | Spirit | 2.52 | 0.22 | 2.14 | 2.89 |
| **Malta** | Anxiety | 6566.50 | 274.41 | 5760.39 | 6853.29 |
|  | Wine | 2.21 | 0.55 | 0.93 | 4.16 |
|  | Beer | 2.70 | 0.33 | 1.74 | 3.07 |
|  | Spirit | 1.89 | 0.26 | 1.50 | 2.51 |
| **Nauru** | Anxiety | 3563.12 | 116.56 | 3282.33 | 3686.007 |
|  | Wine | 0.08 | 0.04 | 0.01 | 0.24 |
|  | Beer | 2.09 | 1.30 | 0.48 | 4.36 |
|  | Spirit | 1.28 | 0.71 | 0.11 | 2.8 |
| **Netherlands** | Anxiety | 6953.92 | 330.46 | 6418.20 | 7324.80 |
|  | Wine | 2.86 | 0.24 | 2.29 | 3.18 |
|  | Beer | 4.67 | 0.42 | 3.95 | 5.2 |
|  | Spirit | 1.80 | 0.37 | 1.32 | 2.47 |
| **New Zealand** | Anxiety | 7405.93 | 261.08 | 6686.64 | 7675.17 |
|  | Wine | 2.74 | 0.32 | 2.11 | 3.23 |
|  | Beer | 4.48 | 1.25 | 3.28 | 7.25 |
|  | Spirit | 1.45 | 0.15 | 1.16 | 1.92 |
| **Norway** | Anxiety | 7180.58 | 183.60 | 6937.38 | 7567.55 |
|  | Wine | 1.75 | 0.52 | 0.91 | 2.3 |
|  | Beer | 2.82 | 0.15 | 2.57 | 3.17 |
|  | Spirit | 1.11 | 0.12 | 0.96 | 1.35 |
| **Oman** | Anxiety | 4649.72 | 136.24 | 4380.46 | 4858.46 |
|  | Wine | 0.13 | 0.00 | 0.00 | 0.03 |
|  | Beer | 0.22 | 0.05 | 0.12 | 0.31 |
|  | Spirit | 0.28 | 0.03 | 0.23 | 0.38 |
| **Poland** | Anxiety | 3423.54 | 78.41 | 3254.83 | 3498.48 |
|  | Wine | 1.211 | 0.35 | 0.82 | 1.9 |
|  | Beer | 4.42 | 1.36 | 2.02 | 5.9 |
|  | Spirit | 3.69 | 0.82 | 2.07 | 5.15 |
| **Portugal** | Anxiety | 8688.84 | 134.37 | 8367.51 | 8975.78 |
|  | Wine | 6.80 | 1.0 | 5.16 | 9.33 |
|  | Beer | 3.27 | 0.54 | 2.47 | 4.1 |
|  | Spirit | 1.45 | 0.56 | 0.7 | 2.46 |
| **Qatar** | Anxiety | 4436.16 | 264.20 | 4072.54 | 5007.89 |
|  | Wine | 0.08 | 0.00 | 0.57 | 0.09 |
|  | Beer | 0.22 | 0.09 | 0.07 | 0.37 |
|  | Spirit | 0.66 | 0.14 | 0.35 | 1.07 |
| **Saint Kitts and Nevis** | Anxiety | 4763.81 | 594.06 | 4109.38 | 5858.94 |
|  | Wine | 0.84 | 0.26 | 0.41 | 1.28 |
|  | Beer | 3.19 | 0.54 | 2.17 | 3.99 |
|  | Spirit | 3.78 | 1.38 | 1.31 | 6.31 |
| **Saudi Arabia** | Anxiety | 4471.19 | 269.04 | 4160.64 | 4956.56 |
|  | Wine | 0.00 | 0.00 | 0.00 | 0.00 |
|  | Beer | -0.05 | 0.12 | -0.25 | 0.21 |
|  | Spirit | 0.06 | 0.04 | -0.00 | 0.13 |
| **Seychelles** | Anxiety | 3824.86 | 89.28 | 3616 | 3909.63 |
|  | Wine | 0.63 | 0.30 | 0.22 | 1.49 |
|  | Beer | 4.08 | 1.00 | 2.72 | 6.32 |
|  | Spirit | 1.82 | 1.55 | 0.40 | 4.62 |
| **Slovakia** | Anxiety | 3802.91 | 89.98 | 3611.85 | 3886.65 |
|  | Wine | 2.07 | 0.27 | 1.56 | 2.83 |
|  | Beer | 4.48 | 1.05 | 3.19 | 6.4 |
|  | Spirit | 4.58 | 0.44 | 3.65 | 5.57 |
| **Slovenia** | Anxiety | 3801.61 | 61.37 | 3659.46 | 3879.64 |
|  | Wine | 5.44 | 1.19 | 3.86 | 8.03 |
|  | Beer | 4.833 | 0.61 | 3.07 | 5.73 |
|  | Spirit | 1.43 | 0.59 | 0.8 | 2.56 |
| **South Korea** | Anxiety | 3574.78 | 143.08 | 3401.62 | 3888.88 |
|  | Wine | 0.08 | 0.06 | 0.01 | 0.19 |
|  | Beer | 2.10 | 0.24 | 1.72 | 2.48 |
|  | Spirit | 0.68 | 0.79 | 0.09 | 2.7 |
| **Spain** | Anxiety | 5094.37 | 435.12 | 4435.04 | 5694.89 |
|  | Wine | 3.59 | 1.01 | 1.21 | 5.1 |
|  | Beer | 4.29 | 0.39 | 3.77 | 5.15 |
|  | Spirit | 2.85 | 0.45 | 2.16 | 3.92 |
| **Sweden** | Anxiety | 4987.98 | 28.30 | 4944.35 | 5029.17 |
|  | Wine | 2.65 | 0.70 | 1.65 | 3.5 |
|  | Beer | 3.04 | 0.55 | 2.57 | 4.15 |
|  | Spirit | 1.29 | 0.34 | 0.99 | 2.1 |
| **Switzerland** | Anxiety | 7132.18 | 193.34 | 6890.92 | 7410.44 |
|  | Wine | 5.28 | 0.67 | 4.23 | 6.5 |
|  | Beer | 3.39 | 0.30 | 3.04 | 4.1 |
|  | Spirit | 1.85 | 0.11 | 1.65 | 2.17 |
| **Trinidad and Tobago** | Anxiety | 3774.28 | 149.88 | 3442.88 | 3904.10 |
|  | Wine | 0.14 | 0.06 | 0.04 | 0.31 |
|  | Beer | 2.40 | 0.62 | 0.76 | 2.92 |
|  | Spirit | 2.70 | 0.55 | 1.39 | 3.94 |
| **United Arab Emirates** | Anxiety | 4413.83 | 161.31 | 4019.34 | 4680.19 |
|  | Wine | 0.13 | 0.04 | 0.06 | 0.24 |
|  | Beer | 0.45 | 0.23 | 0.2 | 0.99 |
|  | Spirit | 1.80 | 0.64 | 0.99 | 3.29 |
| **United Kingdom** | Anxiety | 4622.74 | 125.50 | 4304.13 | 4791.34 |
|  | Wine | 2.90 | 0.63 | 1.8 | 3.60 |
|  | Beer | 4.51 | 0.84 | 3.3 | 5.62 |
|  | Spirit | 2.11 | 0.22 | 1.75 | 2.52 |
| **United States** | Anxiety | 6139.27 | 537.32 | 5453.26 | 7189.4 |
|  | Wine | 1.35 | 0.21 | 1.06 | 1.67 |
|  | Beer | 4.48 | 0.28 | 3.97 | 5.07 |
|  | Spirit | 2.72 | 0.29 | 2.35 | 3.29 |
| **Uruguay** | Anxiety | 5223.48 | 92.71 | 5115.15 | 5389.11 |
|  | Wine | 3.91 | 0.54 | 2.86 | 4.87 |
|  | Beer | 1.62 | 0.31 | 0.85 | 2.02 |
|  | Spirit | 1.03 | 0.38 | 0.58 | 1.93 |

Source: Authors' compilation.
