# Supplementary material for: Evaluating the synergy: anxiety prevalence and alcohol consumption patterns in high-income countries using Granger causality analysis
Source: BMC Public Health. 2025 Jan 20;25:220. doi: 10.1186/s12889-025-21402-6 (PMC11744946; doi:10.1186/s12889-025-21402-6)
Supplement: Supplementary file 3 — Additional file 3. S3 Appendix. LLC unit root test [file 12889_2025_21402_MOESM3_ESM.docx]

**S3 Appendix. LLC unit root test.**

| **Country** | **Anxiety** | **dAnxiety** | **ddAnxeity** | **dddAnxeity** | **Wine** | **dWine** | **Beer** | **dBeer** | **Spirit** | **dSpirit** |
| --- | --- | --- | --- | --- | --- | --- | --- | --- | --- | --- |
| **Africa** |  |  |  |  |  |  |  |  |  |  |
| Seychelles | -3.723*** | - | - | - | -2.989** | - | - | -3.730*** | - | -3.730*** |
| **Asia** |  |  |  |  |  |  |  |  |  |  |
| Bahrain | - | - | - | -2.625** | -3.730*** | - | - | -3.730*** | - | -3.730*** |
| Brunei | -3.730*** | - | - | - | -2.896** | - | -2.989** | - | -2.989** | - |
| Israel | - | - | - | -2.997** | - | -3.730*** | - | -3.730*** | - | -3.730*** |
| Japan | - | - | - | -3.743*** | - | -2.992** | - | -3.730*** | - | -3.730*** |
| Oman | - | -3.730*** | - | - | - | -3.730*** | - | -3.730*** | - | -3.730*** |
| Qatar | - | - | -2.994** | - | -3.730*** | - | - | -3.730** | -2.989** | - |
| Saudi Arabia | - | - | -2.994** | - | - | -3.730*** | - | -3.730*** | - | -3.730*** |
| South Korea | - | - | -2.994** | - | - | -3.730*** | - | -3.730*** | -2.989** | - |
| United Arab Emirates | -2.625* | - | - | - | -2.625* | - | - | -2.992** | - | -3.370*** |
| **Europe** |  |  |  |  |  |  |  |  |  |  |
| Andorra | - | -3.730*** | - | - | - | -3.730*** | - | -3.730*** | - | -3.730*** |
| Austria | - | -2.625* | - | - | - | -3.730*** | - | -3.730*** | - | -3.730*** |
| Belgium | - | - | - | -3.743*** | - | -3.730*** | - | -3.730*** | -3.730*** | - |
| Croatia | - | - | -.2.992** | - | - | -3.730*** | - | -3.730*** | -3.730*** | - |
| Cyprus | - | - | -2.653* | - | - | -3.730*** | - | -3.730*** | -3.730*** | - |
| Czechia | -3.723*** | - | - | - | - | -7.937*** | - | -3.730*** | - | -3.730*** |
| Denmark | - | - | - | -3.911*** | -2.625* | - | - | -3.730*** | - | -3.730** |
| Estonia | - | - | -3.736*** | - | - | -3.730*** | - | -3.730*** | - | -3.730*** |
| Finland | - | -2.992** | - | - | - | -3.730*** | - | -3.730*** | - | -3.730*** |
| France | - | - | - | -2.997** | - | -3.730*** | -2.625* | - | - | -3.730*** |
| Germany | - | - | - | -2.629*** | -3.723*** | - | - | -3.730*** | - | -3.730*** |
| Greece | -2.625* | - | - | - | - | -3.730*** | - | -3.730*** | - | -2.992** |
| Hungary | -2.994** | - | - | - | - | -3.730*** | -3.730*** | - | -2.989** | - |
| Iceland | - | - | -2.994** | - | - | -3.730*** | - | -3.730*** | -2.989** | - |
| Ireland | - | - | - | -3.743*** | - | -3.730*** | - | -3.730*** | - | -3.730*** |
| Italy | - | - | - | -3.743*** | - | -3.730*** | - | -3.730*** | - | -3.730*** |
| Latvia | -2.989** | - | - | - | -3.723* | - | - | -3.730*** | - | -3.730*** |
| Lithuania | -2.989** | - | - | - | - | -3.730*** | - | -2.992** | - | -3.730*** |
| Luxembourg | -3.723*** | - | - | - | -2.625* | - | - | -3.730*** | - | -3.730*** |
| Malta | - | - | - | -3.743*** | -2.989** | - | - | -3.730*** | - | -3.730*** |
| Netherlands | - | - | - | -3.743*** | -2.625* | - | - | -3.730*** | - | -3.730*** |
| Norway | -2.984** | - | - | - | - | -3.730*** | - | -3.730*** | - | -3.730*** |
| Poland | -3.723*** | - | - | - | - | -3.730*** | - | -3.730*** | - | 3.730*** |
| Portugal | - | - | 0.0354** | - | - | -3.730*** | - | -3.730*** | - | 3.730*** |
| Slovakia | -3.723*** | - | - | - | - | -3.730*** | - | -3.370*** | -2.989** | - |
| Slovenia | -2.892** | - | - | - | -2.989** | - | -3.723*** | - | - | -3.730*** |
| Spain | - | - | - | -3.743*** | - | -3730*** | - | -3.730*** | - | -3.730*** |
| Sweden | - | - | - | -3.743*** | - | -3.730*** | - | -3.730*** | -2.989** | - |
| Switzerland | - | - | -2.628* | - | - | -3.730*** | - | -3.730*** | -2.989** | - |
| United Kingdom | -3.723*** | - | - | - | - | -3.730** | - | -2.992** | - | -3.730*** |
| **North America** | |  |  |  |  |  |  |  |  |  |
| Antigua and Barbuda | - | - | -2.286** | - | - | -3.730*** | - | -3.730*** | - | - |
| Bahamas | - | - | -2.628* | - | -2.989** | - | - | -3.730*** | - | -3.730*** |
| Barbados | - | - | - | -3.743*** | - | -3.730*** | - | -3.730*** | - | -3.730*** |
| Canada | - | - | - | -2.984** | - | -3.730*** | - | -3.730*** | - | -3.730*** |
| Saint Kitts and Nevis | - | - | -2.286** | - | - | -3.370*** | - | -3.370*** | - | -3.370*** |
| United States | - | - | - | -3.743*** | - | -3.730*** | - | -3.730*** | - | -3.730*** |
| **Oceania** | |  |  |  |  |  |  |  |  |  |
| Australia | - | - | -2.286** | - | - | -2.989** | -2.989** | - | - | -3.730*** |
| Nauru | - | - | -2.994** | - | -3.730*** | - | - | -3.730*** | - | 3.730*** |
| New Zealand | - | - | -3.723*** | - | - | -3.730*** | - | -3.730*** | -3.730*** | - |
| **South America** | |  |  |  |  |  |  |  |  |  |
| Chile | - | - | - | -2.989** | -3.730*** | - | - | -3.370*** | - | -3.370*** |
| Trinidad Tobago | -3.723*** | - | - | - | - | -3.730*** | - | -3.730*** | -2.989** | - |
| Uruguay | - | - | - | -3.743*** | - | -3.730*** | - | -3.730*** | - | -3.730*** |

Note: * denotes significant at the 10% level, ** at the 5% level, and *** at the 1% level. dAnxiety = first difference of anxiety; ddAnxiety = second difference of anxiety; dddAnxiety = third difference of anxiety; dWine = first difference of wine; dBeer = first difference of beer; dSpirit = first difference of spirits.
