# Supplementary material for: Evaluating the synergy: anxiety prevalence and alcohol consumption patterns in high-income countries using Granger causality analysis
Source: BMC Public Health. 2025 Jan 20;25:220. doi: 10.1186/s12889-025-21402-6 (PMC11744946; doi:10.1186/s12889-025-21402-6)
Supplement: Supplementary file 4 — Additional file 4. S4 Appendix. High-income countries analysis for anxiety and wine from Panel Granger causality [file 12889_2025_21402_MOESM4_ESM.docx]

**S4 Appendix. High-income countries analysis for anxiety and wine from Panel Granger causality.**

| **Country** | **Anxiety to Wine** | **Wine to Anxiety** | **Anxiety - Wine** |
| --- | --- | --- | --- |
| **Africa** |  |  |  |
| Seychelles | 21.1480*** | 4.7359 | Anxiety Wine |
| **Asia** |  |  |  |
| Bahrain | 15.2500*** | 51.5720*** | dddAnxiety Wine |
| Brunei | 20.3510*** | 56.6590*** | Anxiety Wine |
| Israel | 13.8560*** | 8.2031* | dddAnxiety dWine |
| Japan | 10.3150** | 2.5179 | dddAnxiety dWine |
| Oman | 25.9140*** | 42.8790*** | dAnxiety Wine |
| Qatar | 9.9167** | 55.8185 | ddAnxiety dWine |
| Saudi Arabia | 8.1739* | 9.7624** | ddAnxiety dWine |
| South Korea | 1.3920 | 1.1989 | ddAnxiety dWine |
| United Arab Emirates | 1.4430 | 5.0480 | Anxiety Wine |
| **Europe** |  |  |  |
| Andorra | 0.8750*** | 7.5286*** | dAnxiety dWine |
| Austria | 4.1860 | 10.4100*** | dAnxiety dWine |
| Belgium | 27.1780*** | 66.8810 | dddAnxiety dWine |
| Croatia | 40.8060*** | 34.5890*** | ddAnxiety dWine |
| Cyprus | 18.8940 | 19.7380 | ddAnxiety dWine |
| Czechia | 32.1950 | 91.6990 | Anxiety dWine |
| Denmark | 4.0663 | 1.4068 | dddAnxiety Wine |
| Estonia | 0.1619 | 2.9652 | ddAnxiety dWine |
| Finland | 4.9541 | 11.9550** | dAnxiety dWine |
| France | 5.1678 | 57.5400*** | dddAnxiety dWine |
| Germany | 16.5170*** | 106.9800*** | dddAnxiety Wine |
| Greece | 17.9070 | 13.8630*** | ddAnxiety dWine |
| Hungary | 0.161 | 0.023** | Anxiety dWine |
| Iceland | 2.7488 | 6.5560** | ddAnxiety dWine |
| Ireland | 1.7059 | 4.0134 | dddAnxiety dWine |
| Italy | 43.2310*** | 9.7632** | dddAnxiety dWine |
| Latvia | 16.761*** | 4.0274 | Anxiety Wine |
| Lithuania | 17.0830*** | 7.7604 | Anxiety dWine |
| Luxembourg | 2.5019 | 6.1384 | Anxiety dWine |
| Malta | 0.1631 | 0.1607 | dddAnxiety Wine |
| Netherlands | 70.4460 | 25.6720** | dAnxiety Wine |
| Norway | 58.3180** | 12.4100*** | Anxiety dWine |
| Poland | 32.553*** | 2.5936 | Anxiety dWine |
| Portugal | 11.4260 | 13.2120*** | Anxiety dWine |
| Slovakia | 11.0900** | 57.4920*** | Anxiety dWine |
| Slovenia | 13.1700** | 57.3020*** | Anxiety Wine |
| Spain | 1.4812 | 0.2926 | dddAnxiety dWine |
| Sweden | 0.3132 | 1.2867 | dddAnxiety dWine |
| Switzerland | 1.971 | 8.3927*** | ddAnxiety dWine |
| United Kingdom | 7.1914 | 4.9687 | Anxiety Wine |
| **North America** |  |  |  |
| Antigua and Barbuda | 12.5562 | 9.4187 | dddAnxiety dWine |
| Bahamas | 7.5321*** | 27.3620*** | dAnxiety Wine |
| Barbados | 2.9005 | 0.1973 | dddAnxiety dWine |
| Canada | 22.8260*** | 21.3450** | dddAnxiety dWine |
| Saint Kitts and Nevis | 1.8361 | 0.5079 | dddAnxiety Wine |
| United States | 0.0859 | 2.5884 | dddAnxiety dWine |
| **Oceania** |  |  |  |
| Australia | 0.4254 | 3.5332 | ddAnxiety dWine |
| Nauru | 0.8992 | 7.5394** | dAxiety Wine |
| New Zealand | 4.9959 | 18.5500*** | ddAnxiety dWine |
| **South America** |  |  |  |
| Chile | 7.5412*** | 3.9761 | dddAnxiety Wine |
| Trinidad Tobago | 1.1383 | 15.476*** | ddAnxiety dWine |
| Uruguay | 23.419*** | 0.3982 | dddAnxiety dWine |

Note: The characters and represents one-way-right direction and one-way-left direction causal relationship, and represents a bidirectional, no causal relationship, respectively. These arrows are shown in four sizes; very small, small, medium and large. The length is shown as no difference, 1st difference, 2nd difference and 3rd difference in order from the smallest to the largest. The strength of the causal relationship is shown when the differences decrease from high to low. * denotes significant at the 10% level, ** at the 5% level, and *** at the 1% level.
