# Supplementary material for: Evaluating the synergy: anxiety prevalence and alcohol consumption patterns in high-income countries using Granger causality analysis
Source: BMC Public Health. 2025 Jan 20;25:220. doi: 10.1186/s12889-025-21402-6 (PMC11744946; doi:10.1186/s12889-025-21402-6)
Supplement: Supplementary file 5 — Additional file 5. S5 Appendix. High-income countries analysis for anxiety and beer from Panel Granger causality [file 12889_2025_21402_MOESM5_ESM.docx]

**S5 Appendix. High-income countries analysis for anxiety and beer from Panel Granger causality.**

| **Country** | **Anxiety to Beer** | **Beer to Anxiety** | **Anxiety - Beer** |
| --- | --- | --- | --- |
| **Africa** | |  |  |
| Seychelles | 35.6130*** | 1.5206 | Anxiety dBeer |
| **Asia** |  |  |  |
| Bahrain | 32.6210*** | 84.8350*** | dddAnxiety dBeer |
| Brunei | 22.3750** | 58.5890*** | Anxiety Beer |
| Israel | 14.2860*** | 4.4445 | dddAnxiety dBeer |
| Japan | 42.6720*** | 4.4242 | dddAnxiety dBeer |
| Oman | 3.4801 | 13.3670** | dAnxiety dBeer |
| Qatar | 2.7424 | 8.7160* | ddAnxiety dBeer |
| Saudi Arabia | 1.8501 | 1.7257 | ddAnxiety dBeer |
| South Korea | 0.2492 | 0.9480 | ddAnxiety dBeer |
| United Arab Emirates | 3.1360 | 4.5184 | Anxiety dBeer |
| **Europe** |  |  |  |
| Andorra | 2.3071 | 2.0463*** | dAnxiety dBeer |
| Austria | 1.1574* | 6.1085** | dAnxiety dBeer |
| Belgium | 31.3040*** | 93.0320** | dddAnxiety dBeer |
| Croatia | 11.8340*** | 44.4360** | ddAnxiety dBeer |
| Cyprus | 2.9924 | 28.8630*** | ddAnxiety dBeer |
| Czechia | 0.6982 | 1.7318 | Anxiety dBeer |
| Denmark | 1.6007 | 1.1790 | dddAnxiety dBeer |
| Estonia | 2.3702 | 2.3034 | ddAnxiety dBeer |
| Finland | 20.3050*** | 17.666*** | dAnxiety dBeer |
| France | 32.5890*** | 50.1250*** | dddAnxiety Beer |
| Germany | 2.8933 | 63.9990*** | dddAnxiety dBeer |
| Greece | 13.1320*** | 22.1180*** | ddAnxiety dBeer |
| Hungary | 3.9983*** | 0.3231*** | Anxiety Beer |
| Iceland | 0.4041 | 8.0236** | ddAnxiety dBeer |
| Ireland | 4.6738* | 3.6054 | dddAnxiety dBeer |
| Italy | 83.8180*** | 12.0880** | dddAnxiety dBeer |
| Latvia | 64.3210*** | 17.7080*** | Anxiety dBeer |
| Lithuania | 57.6310*** | 4.6146 | Anxiety dBeer |
| Luxembourg | 60.0780* | 3.5590* | Anxiety dBeer |
| Malta | 0.1684 | 0.1719 | dddAnxiety dBeer |
| Netherlands | 5.1360*** | 3.7300 | dAnxiety dBeer |
| Norway | 2.4980*** | 3.7230 | Anxiety dBeer |
| Poland | 19.5300*** | 7.6978 | Anxiety dBeer |
| Portugal | 48.8850* | 13.2120** | dAnxiety dSpirit |
| Slovakia | 20.0550*** | 4.0840 | Anxiety Beer |
| Slovenia | 6.7746 | 78.7580*** | Anxiety Beer |
| Spain | 0.5562 | 0.4827 | dddAnxiety dBeer |
| Sweden | 0.7157 | 0.4260 | dddAnxiety dBeer |
| Switzerland | 0.4036 | 3.1343* | ddAnxiety dBeer |
| United Kingdom | 4.3502 | 2.5127 | Anxiety dBeer |
| **North America** |  |  |  |
| Antigua and Barbuda | 0.2898 | 4.6660 | dddAnxiety dBeer |
| Bahamas | 11.0730*** | 15.2820* | dAnxiety dBeer |
| Barbados | 1.6972 | 0.6522 | dddAnxiety dBeer |
| Canada | 21.0750*** | 6.9402** | dddAnxiety dBeer |
| Saint Kitts and Nevis | 3.4345 | 2.0370 | dddAnxiety dBeer |
| United States | 2.0381 | 1.5688 | dddAnxiety dBeer |
| **Oceania** |  |  |  |
| Australia | 5.7243 | 0.6132 | ddAnxiety Beer |
| Nauru | 1.4769 | 2.4876 | dAnxiety dBeer |
| New Zealand | 57.8820*** | 22.7640*** | ddAnxiety dBeer |
| **South America** |  |  |  |
| Chile | 7.4496** | 10.8360 | dddAnxiety dBeer |
| Trinidad Tobago | 2.0255 | 0.8063 | ddAnxiety Beer |
| Uruguay | 3.1486 | 4.6636* | dddAnxiety dBeer |

Note: The characters and represents one-way-right direction and one-way-left direction causal relationship, and represents a bidirectional, no causal relationship, respectively. These arrows are shown in four sizes; very small, small, medium, and large. The length is shown as no difference, 1st difference, 2nd difference and 3rd difference in order from the smallest to the largest. The strength of the causal relationship is shown when the differences decrease from high to low. * denotes significant at the 10% level, ** at the 5% level, and *** at the 1% level.
