# Supplementary material for: Evaluating the synergy: anxiety prevalence and alcohol consumption patterns in high-income countries using Granger causality analysis
Source: BMC Public Health. 2025 Jan 20;25:220. doi: 10.1186/s12889-025-21402-6 (PMC11744946; doi:10.1186/s12889-025-21402-6)
Supplement: Supplementary file 6 — Additional file 6. S6 Appendix. High-income countries analysis for anxiety and spirits from Panel Granger causality [file 12889_2025_21402_MOESM6_ESM.docx]

**S6 Appendix. High-income countries analysis for anxiety and spirits from Panel Granger causality.**

| **Country** | **Anxiety to Spirit** | **Spirit to Anxiety** | **Anxiety - Spirit** |
| --- | --- | --- | --- |
| **Africa** |  |  |  |
| Seychelles | 9.2792* | 4.3049 | Anxiety dSpirit |
| **Asia** |  |  |  |
| Bahrain | 313.6000*** | 115.7900*** | dddAnxiety dSpirit |
| Brunei | 131.9100*** | 29.2660*** | Anxiety Spirit |
| Israel | 17.673*** | 12.462** | dddAnxiety dSpirit |
| Japan | 38.6540*** | 8.2169* | dddAnxiety dSpirit |
| Oman | 0.9877 | 39.0720*** | dAxiety dSpirit |
| Qatar | 17.1940*** | 2.4587 | ddAnxiety Spirit |
| Saudi Arabia | 24.7890*** | 7.1792 | ddAnxiety Spirit |
| South Korea | 0.0532 | 1.2167 | dAnxiety Spirit |
| United Arab Emirates | 5.3830 | 11.2030** | Anxiety dSpirit |
| **Europe** |  |  |  |
| Andorra | 0.5955 | 0.3486*** | dAnxiety dSpirit |
| Austria | 3.6848** | 7.7521*** | dAnxiety dSpirit |
| Belgium | 9.0294** | 84.9830 | dddAnxiety Spirit |
| Croatia | 2.0347*** | 103.2300*** | ddAnxiety Spirit |
| Cyprus | 2.1216 | 0.8407 | ddAnxiety Spirit |
| Czechia | 0.7397 | 3.4152 | Anxiety dSpirit |
| Denmark | 0.0126 | 1.2906 | dddAnxiety dSpirit |
| Estonia | 2.9815 | 3.1346 | ddAnxiety Spirit |
| Finland | 4.1298 | 13.6280*** | dAnxiety dSpirit |
| France | 26.2080*** | 57.4750*** | dddAnxiety dSpirit |
| Germany | 0.8447 | 111.3500*** | dddAnxiety dSpirit |
| Greece | 8.3494*** | 23.9750*** | ddAnxiety Spirit |
| Hungary | 1.3111*** | 3.5171** | Anxiety Spirit |
| Iceland | 29.0360*** | 5.9742 | ddAnxiety Spirit |
| Ireland | 0.6589 | 3.9785 | dddAnxiety dSpirit |
| Italy | 22.1410*** | 6.1460 | dddAnxiety dSpirit |
| Latvia | 45.1140*** | 10.3230** | Anxiety dSpirit |
| Lithuania | 11.7190** | 17.6430*** | Anxiety dSpirit |
| Luxembourg | 17.7630*** | 15.3720 | Anxiety dSpirit |
| Malta | 2.3241 | 0.6025 | dddAnxiety dSpirit |
| Netherlands | 50.3223 | 58.5652 | dAnxiety Spirit |
| Norway | 9.3454*** | 6.5918*** | Anxiety dSpirit |
| Poland | 32.6840*** | 15.6940*** | Anxiety dSpirit |
| Portugal | 0.4041 | 16.4440*** | dAnxiety dSpirit |
| Slovakia | 12.7730** | 19.6230*** | Anxiety dSpirit |
| Slovenia | 6.7710 | 15.5110*** | Anxiety Spirit |
| Spain | 1.4166 | 0.4382 | dddAnxiety dSpirit |
| Sweden | 2.0054 | 1.0491 | dddAnxiety dSpirit |
| Switzerland | 0.5283 | 2.1628 | ddAnxiety Spirit |
| United Kingdom | 33.6110*** | 2.2482 | Anxiety dSpirit |
| **North America** |  |  |  |
| Antigua and Barbuda | 3.7360*** | 3.9140 | dddAnxiety dSpirit |
| Bahamas | 4.2520 | 14.8630 | dAnxiety dSpirit |
| Barbados | 4.4667 | 46.1920** | dddAnxiety dSpirit |
| Canada | 5.3273** | 5.1366** | dddAnxiety dSpirit |
| Saint Kitts and Nevis | 1.8266 | 0.0770 | dddAnxiety dSpirit |
| United States | 2.0757 | 0.1231 | dddAnxiety dSpirit |
| **Oceania** |  |  |  |
| Australia | 0.0422 | 0.0090 | ddAnxiety dSpirit |
| Nauru | 2.2432*** | 16.3130 | dAnxiety dSpirit |
| New Zealand | 8.5321* | 58.3000*** | ddAnxiety Spirit |
| **South America** |  |  |  |
| Chile | 58.1750*** | 5.6638*** | dddAnxiety Spirit |
| Trinidad Tobago | 1.5763 | 0.0874 | ddAnxiety Spirit |
| Uruguay | 1.2970 | 8.8477** | dddAnxiety dSpirit |

Note: The characters and represents one-way-right direction and one-way-left direction causal relationship, and represents a bidirectional, no causal relationship, respectively. These arrows are shown in four sizes: very small, small, medium, and large. The length is shown as no difference, 1st difference, 2nd difference and 3rd difference in order from the smallest to the largest. The strength of the causal relationship is shown when the differences decrease from high to low. * denotes significant at the 10% level, ** at the 5% level, and *** at the 1% level.
